# Efficacy of a Mindfulness-Based Mobile Application: a Randomized Waiting-List Controlled Trial

**DOI:** 10.1007/s12671-017-0761-7

**Published:** 2017-06-21

**Authors:** Arnold A. P. van Emmerik, Fieke Berings, Jaap Lancee

**Affiliations:** 1 Department of Clinical Psychology, Faculty of Social and Behavioural Sciences, University of Amsterdam, Nieuwe Achtergracht 129-B, 1018 WS Amsterdam, The Netherlands; 2 Department of Quality and Innovation, Health Insurance Company VGZ, Nieuwe Stationsstraat 12, 6811 KS Arnhem, The Netherlands

**Keywords:** Mindfulness, E-health, M-health, Intervention, Quality of life, Psychiatry

## Abstract

Although several hundreds of apps are available that (cl)aim to promote mindfulness, only a few methodologically sound studies have evaluated the efficacy of these apps. This randomized waiting-list controlled trial therefore tested the hypothesis that one such app (the *VGZ Mindfulness Coach*) can achieve immediate and long-term improvements of mindfulness, quality of life, general psychiatric symptoms, and self-actualization. One hundred ninety-one experimental participants received the VGZ Mindfulness Coach, which offers 40 mindfulness exercises and background information about mindfulness without any form of therapeutic guidance. Compared to 186 control participants, they reported large (Cohen’s *d* = 0.77) and statistically significant increases of mindfulness after 8 weeks and small-to-medium increases of the Observing, Describing, Acting with awareness, Nonjudging, and Nonreactivity mindfulness facets as measured with the Five Facet Mindfulness Questionnaire (Cohen’s *d* = 0.66, 0.26, 0.49, 0.34, and 0.43, respectively). Also, there were large decreases of general psychiatric symptoms (GHQ-12; Cohen’s *d* = −0.68) and moderate increases of psychological, social, and environmental quality of life (WHOQOL-BREF; Cohen’s *d* = 0.38, 0.38, and 0.36, respectively). Except for social quality of life, these gains were maintained for at least 3 months. We conclude that it is possible to achieve durable positive effects on mindfulness, general psychiatric symptoms, and several aspects of quality of life at low costs with smartphone apps for mindfulness such as the VGZ Mindfulness Coach.

**Electronic supplementary material:**

The online version of this article (doi:10.1007/s12671-017-0761-7) contains supplementary material, which is available to authorized users.

## Introduction

The origins of the current clinical and research interest in mindfulness-based interventions (MBIs) can be traced back to a landmark study by Jon Kabat-Zinn (1982), who demonstrated the beneficial effects of the “Stress Reduction and Relaxation Program” on pain, mood, and psychiatric symptomatology in chronic pain patients. The interest in MBIs has exponentially increased ever since. Probably the first empirical review of MBIs was conducted by Baer (2003). In a meta-analysis of 21 outcome studies, MBIs were effective across a range of clinical and nonclinical samples (*d* = 0.59) and particularly large effects were found for psychological- and psychopathological-dependent variables (e.g., *d* = 0.86 for depression).

The number of MBI outcome studies has rapidly increased ever since, and various (overlapping) reviews and meta-analyses have subsequently appeared (e.g., Bohlmeijer et al. 2010; Eberth and Sedlmeier 2012; Hofmann et al. 2010; Grossman et al. 2004; Sedlmeier et al. 2012). Generally (but not equivocally, i.e., Toneatto and Nguyen 2007), these meta-analyses corroborate the beneficial effects of MBIs across a wide variety of samples and somatic-, psychological-, and psychopathological-dependent variables. With regard to mindfulness as a dependent variable, Eberth and Sedlmeier (2012) and Sedlmeier et al. (2012) found medium effect sizes of *r* = 0.34 and *r* = 0.28, respectively.

There are indications that underlying the general efficacy of MBIs is a more fine-grained pattern of relationships between MBI effect sizes and study characteristics such as type of sample, type of MBI, methodological quality, and type of dependent variable.

Regarding type of sample, for instance, Baer (2003) found similar effect sizes for nonclinical samples (students and nonclinical volunteers; *d* = 0.92) and axis I disorder patients (anxiety, depression, and binge-eating patients; *d* = 0.96), but considerably smaller effect sizes for medical patients (fibromyalgia, psoriasis, and cancer patients; *d* = 0.55) and chronic pain patients (*d* = 0.37).

Regarding type of MBI, Eberth and Sedlmeier (2012) found that mindfulness alone showed significantly (but not dramatically) larger effects on mindfulness measures (*r* = 0.37) than the comprehensive Mindfulness-Based Stress Reduction (MBSR) programs (*r* = 0.29). Tentative explanations for this difference suggested by the authors include that the latter programs also offer nonmindfulness components such as psychoeducation on stress and self-care and that the name of these programs may evoke expectations of positive effects (and hence actual effects) on other outcome variables (i.e., stress) than on mindfulness per se. While the general efficacy of MBIs is thus convincingly demonstrated to date, their effect sizes seem to depend on (interactions of) these and other study characteristics in intricate ways.

As with other evidence-based interventions, most MBIs strongly rely on face-to-face delivery, with dissemination limitations as a result. Put simply, not enough therapists are available to provide face-to-face MBIs, and not all patients are willing or able to attend face-to-face MBIs. Also, at least in the Netherlands, MBIs are not covered by most health insurance plans. Online (e-health) or smartphone-based (m-health) treatments may help to overcome these problems by providing, at little or no cost, treatment at the patients’ chosen time and place. MBIs might be particularly suitable for these treatment modalities because many of their exercises are fairly easy to explain and involve repetitive practicing which in principle can as easily be achieved at home as under the guidance of a therapist.

Over the past years, there has been a surge of attention for e-health and m-health applications (apps) in health care at large. Several meta-analyses indicate that e-health interventions are effective in the treatment of anxiety and depression (Spek et al. 2007) and other health problems (Cuijpers et al. 2008) with treatment effects that are comparable to face-to-face treatment (Cuijpers et al. 2010). Online interventions now also include MBIs (e.g., Cavanagh et al. 2013; Glück and Maercker 2011). Currently, the development of these interventions is increasingly directed towards smartphone apps (m-health). More than 200 apps related to mindfulness exist for Google Android smartphones alone (Plaza et al. 2013).

In the scientific community, MBI apps have received comparatively little attention however. We could identify only two randomized controlled trials (RCTs) of MBI apps. Ly et al. (2014) compared an 8-week MBI to an 8-week behavioral activation program in 81 participants with major depressive disorder. Both interventions were delivered through smartphone apps. The MBI app offered “an 8-week smartphone-based intervention with a *[*sic*]* minimal therapist contact (maximum time of 20 min per participant per week), consisted of a short web-based psychoeducation, and a step-by-step mindfulness practice program, administered via a smartphone application. (…) The text was written especially for the current intervention, with inspiration from the self help book *The Mindful Way Through Depression* by Williams et al. (2007)” (p. 6). They found large, comparable reductions of depression after both interventions which lasted for at least 6 months, but noted that the study lacked a waiting-list control (WLC) condition. Furthermore, participants wrote weekly reflections on “their work and thoughts on the current treatment week” and “received personal feedback on their reflection from their therapist” (p. 6–7). Hence, the self-help MBI was complemented with a limited amount of therapeutic attention.

Howells et al. (2016) compared Headspace’s smartphone-based Take 10 program (https://www.headspace.com), consisting of 10 min of mindfulness practice over ten consecutive days, to a neutral but active control task (the Catch Notes app) in a nonclinical population. They found significant improvements of positive affect and depression, but not of satisfaction with life, social-psychological prosperity, and negative affect. However, it is unknown how long these effects lasted since the study did not include a follow-up test. More importantly, although both Ly et al. (2014) and Howells et al. (2016) found improvements on several clinical outcome variables, they did not measure mindfulness as the alleged therapeutic mechanism underlying these improvements.

In addition to these RCTs of MBI apps, Chittaro and Vianello (2016) recently provided a thorough review of computer-supported mindfulness techniques, and reported an uncontrolled evaluation of the smartphone-based AEON app in the everyday lives of a large community sample of experienced and nonexperienced meditators. The AEON app aims to increase decentering, which is defined as “a state of awareness of internal events, without responding to them with sustained evaluation, attempts to control or suppress them, or respond to them behaviourally” (Wells 2005). Over the 4-week study period, participants reported significant increases of decentering that were comparable for experienced meditators and participants with no or minimal experience with meditation. In an earlier laboratory study, the AEON app compared favorably to two other decentering interventions (i.e., cloud imagery, Wells 2005 and card tossing, Hayes et al. 1994; Leahy 2006) in terms of increased decentering, pleasantness, and ease of use (Chittaro and Vianello 2014).

These early studies confirmed the potential of apps as a means of disseminating MBIs, but more research is needed to corroborate their findings and to remedy some of their methodological limitations. We therefore expanded upon these important studies by evaluating the immediate and long-term efficacy of an MBI app (the *VGZ Mindfulness Coach*) in a waiting-list controlled randomized trial, with mindfulness as a primary outcome variable and without any form of therapeutic guidance in addition to the self-help app. Secondary outcome measures assessed the impact of the app on quality of life, general psychiatric symptomatology, and self-actualization, a common nonclinical outcome variable in many meditation studies (Alexander et al. 1991). We expected that participants who received the VGZ Mindfulness Coach would demonstrate greater improvements on all these outcomes than participants in the WLC condition.

## Method

### Participants

Participants were recruited through a Facebook advertisement of the study by a social media agency, targeted at people who were known to have an interest in mindfulness and spirituality. The advertisement was directed away from people who had previously liked VGZ’s Facebook page, to avoid the inclusion of participants who were already familiar with the VGZ Mindfulness Coach. Eligibility criteria included (a) being 18 years or older, (b) having sufficient fluency in Dutch to complete the research procedures and use the VGZ Mindfulness Coach, and (c) being willing to provide written informed consent prior to their inclusion in the study. The study procedures were approved by the institutional review board of the Faculty of Social and Behavioural Sciences of the University of Amsterdam, and the study has been performed in accordance with the ethical standards laid down in the 1964 Declaration of Helsinki and its later amendments. The study protocol was preregistered at the Netherlands Trial Register (www.trialregister.nl, TC=5001).

For this study, we wanted to have sufficient power (0.80) for detecting a small effect (*f* ^2^ = 0.10). Based on a repeated measures ANOVA test, this would require a total sample size of at least 200 participants (*p* < 0.05; two-tailed). We anticipated a large dropout rate of 50% for this online study, and therefore aimed to include 400 participants. We were able to include a slightly lower number of participants (*n* = 377), but, since the dropout rate was lower than expected, nevertheless retained enough posttest completers (*n* = 221).

### Procedure

Participants who provided informed consent and met the eligibility criteria were included in the study and were randomly allocated to the experimental or WLC condition. Randomization followed a computer-generated block randomization (blocks of 50) and was carried out by an independent research assistant who was not involved in the statistical analysis or in writing up the study. Participants and researchers were not blinded to the allocated condition.

In the experimental condition, participants were directed to a website where they could download the VGZ Mindfulness Coach from the App Store (https://itunes.apple.com/nl/app/vgz-mindfulness-coach/id779531500?mt=8) or Google Play Store (https://play.google.com/store/apps/details?id=nl.vgz.mindfulness). The study measures were administered before randomization (baseline), 8 weeks after baseline (posttest), and 20 weeks after baseline (follow-up). The 8-week interval between baseline and posttest allowed participants in the experimental condition ample time to complete the 5-week program (see Intervention). Since participants in the WLC condition were offered the VGZ Mindfulness Coach immediately upon completion of the posttest, the follow-up was only conducted in the experimental condition. Participants received no financial or other forms of compensation.

The VGZ Mindfulness Coach is currently available for iOS and Android platforms and was developed by the Health Insurance Company VGZ, which is the second largest health insurance company in the Netherlands, with more than 4,000,000 clients. As shown in Table 1, the app offers 40 audio exercises, including but not limited to breathing exercises, attention exercises, body scan exercises, guided meditation exercises, visualization exercises, mantra exercises, and yoga exercises. It also offers the possibility of a 5-week program of 25 preselected audio exercises, as well as background information on meditation and mindfulness and the various exercises. The exercises were drawn from existing mindfulness programs of Jon Kabat-Zinn and Edel Maex, a Belgian psychiatrist, Zen teacher, and prolific writer on mindfulness. The digitalization of these exercises was directed by a team including an expert psychologist and an experienced mindfulness trainer (Marijke Will, personal communication, September 29, 2016). Exercises can be selected using filters for their length (3–37 min), aim (resting, clarity), and expected setting (public transport, work, home) and optionally appear directly in the user’s agenda app.

**Table 1:** Overview of exercises and 5-week program offered by the VGZ Mindfulness Coach

Full list of exercises	Duration	Exercises in 5-week program	Duration
Sitting (down)	3:05	Week 1	
Attention for the breath	10:04	Relaxation exercise	2:36
Attention for perception	1:51	Walking	3:13
Breathing exercise	11:57	Breathing exercise	11:57
Phone calls	2:28	Introduction to visualization	3:22
Body scan	36:55	A new sound	4:39
Brief body scan	11:24		
Chakra	6:28	Week 2	
3-min breathing space	4:38	Brief concentration exercise	13:06
Drinking	2:57	Brief body scan	11:24
A new sound	4:39	Mantra	7:05
Eating	2:18	Long visualization	8:57
Time for moving	2:02	Body scan	36:55
Time for your body	2:05		
Focus: within your mind	13:55	Week 3	
Guided meditation	8:33	Guided meditation	8:33
Watching	2:44	Sitting (down)	3:05
Brief concentration exercise	13:06	Mindfulness: your thoughts	6:03
Brief meditation exercise	2:12	Body scan	36:55
Brief meditation exercise: grounding	5:16	Visualization	4:51
Walking meditation	10:05		
Walking	3:13	Week 4	
Introduction to visualization	3:22	Time for moving	2:02
Mantra	7:05	Walking meditation	7:27
Mantra walking meditation	11:36	Chakra	6:28
Meditation on music	6:31	Body scan	36:55
Walking meditation	7:27	Guided meditation	8:33
Mindful body meditation	12:32		
Mindfulness: your thoughts	6:03	Week 5	
Mindfulness: your thoughts on…	6:53	3-min breathing space	4:38
Relaxation exercise	2:36	Resting body and mind	4:27
Resting body and mind	4:27	Attention for perception	1:51
Standing	3:42	Brief meditation exercise	2:12
Walking the stairs	1:38	Body scan	36:55
Long visualization	8:57		
Visualization	4:51		
Waiting	2:59		
Yantra	12:04		
Yoga meditation	10:01		
Sitting posture	1:28		

The VGZ Mindfulness Coach is a self-help intervention without any form of automated or therapist-provided guidance or feedback. In our study, participants in the experimental condition were gently encouraged to decide upon the optional 5-week program, and received weekly automated, nonindividualized e-mails to promote their use of the app. The app offers information on how to navigate the various exercises and options of the app (e.g., the filters for length, aim, and setting), but other than the suggestion to follow the 5-week program and the standardized e-mail reminders, participants received no instructions on how the app should be used. The study was conducted completely online, and except for a few e-mails concerning technical difficulties of some participants, there was no contact with participants outside the pre-planned, standardized research procedures.

### Measures

#### Primary Outcome Measure

Mindfulness was assessed with a Dutch version of the Five Facet Mindfulness Questionnaire (FFMQ; Baer et al. 2006; De Bruin et al. 2012). The FFMQ consists of 39 items (present pretest Cronbach’s *α* = 0.91) that are rated 1 (never or very rarely true) to 5 (very often or always true) and reflect five facets of mindfulness: Observing (8 items, present pretest Cronbach’s *α* = 0.79), Describing (8 items, present pretest Cronbach’s *α* = 0.91), Acting with awareness (8 items, present pretest Cronbach’s *α* = 0.86), Nonjudging (8 items, present pretest Cronbach’s *α* = 0.88), and Nonreactivity (7 items, present pretest Cronbach’s *α* = 0.80). The FFMQ total score ranges between 39 and 195 and its subscale scores between 8 and 40 (or 7 and 35 for the Nonreactivity subscale), with higher scores indicating higher levels of mindfulness. The total scale and subscales demonstrated comparable internal consistency at posttest (Cronbach’s *α* = 0.82 to 0.94) and follow-up (Cronbach’s *α* = 0.75 to 0.94). In previous work with meditating and nonmeditating samples, internal consistency was good for the total scale (meditating sample: *α* = 0.90; nonmeditating sample: *α* = 0.85) and adequate to good for the subscales (meditating sample: *α* = 0.72 to 0.89; nonmeditating sample: *α* = 0.70 to 0.87) (De Bruin et al. 2012).

#### Secondary Outcome Measures

Quality of life was measured with a Dutch version of the World Health Organization Quality of Life assessment, short version (WHOQOL-BREF; Trompenaars et al. 2005; WHOQOL-Group 1998). The WHOQOL-BREF consists of two questions that measure overall quality of life and general health, respectively, and 24 questions that measure four quality-of-life domains, including Physical health (7 items, present pretest Cronbach’s *α* = 0.66), Psychological health (6 items, present pretest Cronbach’s *α* = 0.52), Social relationships (3 items, present pretest Cronbach’s *α* = 0.60), and Environment (8 items, present pretest Cronbach’s *α* = 0.68). Questions are rated on five-point Likert scales with answer categories tailored to each question. The WHOQOL-BREF domain scores range between 7 and 35 (Physical health), 6 and 30 (Psychological health), 3 and 15 (Social relationships), and 8 and 40 (Environment), with higher scores indicating higher quality of life. In the present study, the subscales demonstrated slightly better internal consistency at posttest (Cronbach’s *α* = 0.71 to 0.84) and comparable internal consistency at follow-up (Cronbach’s *α* = 0.53 to 0.69). In previous studies, the subscales have demonstrated comparable internal consistency in Dutch psychiatric outpatients (Cronbach’s *α* = 0.66 to 0.80) (Trompenaars et al. 2005) and in a mixed international sample (Cronbach’s *α* = 0.66 to 0.84) (WHOQOL-Group 1998).

General psychiatric symptomatology was assessed with a Dutch version of the General Health Questionnaire-12 (GHQ-12; Goldberg and Williams 1988; Koeter and Ormel 1991). The GHQ-12 consists of 12 items that are rated on four-point Likert scales that are rated 0 to 3, with answer categories tailored to each item. The GHQ-12 total score ranges between 0 and 36, with higher scores indicating higher levels of general psychiatric symptomatology. In the present study, Cronbach’s *α* was 0.89 at pretest and 0.92 and 0.86 at posttest and follow-up, respectively. The GHQ-12 has shown comparable internal consistency in several previous studies (Cronbach’s *α* = 0.82 to 0.90) (McDowell 2006, p. 265).

Self-actualization was assessed with a Dutch version (Kamphuis & van Emmerik, in preparation) of the Short Index of Self-Actualization (SISA; Jones and Crandall 1986). The SISA consists of 15 items that are rated 1 (disagree) to 4 (agree), with higher scores indicating higher levels of self-actualization. In the present study, Cronbach’s *α* was 0.73 at pretest and 0.80 and 0.78 at posttest and follow-up, respectively, which is comparable to or better than the internal consistency of the original English version of the SISA in students (Cronbach’s *α* = 0.65) (Jones and Crandall 1986).

Finally, participants completed a number of questions that assessed their general satisfaction with the VGZ Mindfulness Coach (including such aspects as usability, quality of the voice-over, and clarity and usefulness of content) and the number of weeks and frequency of use of the VGZ Mindfulness Coach. In addition, we collected demographic data (age, sex, relationship status, and educational level), and asked participants whether they currently practiced mindfulness or other forms of meditation. All measurements and data collection took place using the Qualtrics online research platform (www.qualtrics.com).

### Data Analyses

Data were analyzed using IBM SPSS Statistics, version 22 for Windows and MLwin, version 2.30. Since we did not collect adherence data, we were unable to distinguish between study dropouts and intervention dropouts. Participants were therefore classified as dropouts if they failed to complete our primary outcome measure (FFMQ) at posttest or follow-up. All statistical tests were two-tailed.

First, chi-squares were used to determine whether there was a significant association between study condition and dropout rate at posttest. Second, chi-squares and independent-samples *t* tests were used to evaluate differences between completers and dropouts at posttest (in each study condition) or follow-up (in the experimental condition) on the demographic, meditation (currently practicing mindfulness or other forms of meditation), and outcome variables at baseline. Third, chi-squares and independent-samples *t* tests were used to evaluate differences between study conditions on these variables at baseline. Fourth, we used multilevel regression analysis to test for within-group (time) and between-group (time × condition) effects. Multilevel regression analysis was used because this is an intention-to-treat analysis in which all the data can remain in the analyses (Hox 2002). Variables that predicted dropout or showed between-group differences at baseline were used as covariates in the analyses. We ran the analyses on the intention-to-treat and on the completer sample.

Fifth, because attrition could have influenced the posttest scores and in turn our estimates of Cohen’s *d*, we imputed the missing values with a predictive mean matching procedure based on the missing at random assumption (Sterne et al. 2009). Using multiple imputation, we imputed all cases that did not respond to the posttest or follow-up by generating ten separate datasets and replaced the missing values with the mean of these ten sets. Sixth, within-group Cohen’s *d* effect sizes were calculated with (*M*_post_ − *M*_pre_)/*SD*_pooled_. Between-group Cohen’s *d* effect sizes were calculated from the difference in change scores between the study conditions divided by the pooled standard deviation of these change scores. In the intention-to-treat sample, these Cohen’s *d* effect sizes were estimated on the imputed sample.

## Results

### Dropout

Of the 377 participants who met our eligibility criteria and completed the baseline assessment, 191 (50.7%) were allocated to the experimental condition and 186 (49.3%) were allocated to the WLC condition. Across study conditions, 221 (58.6%) completed the posttest assessment. A significant association was found between study condition and dropout rate at posttest (*χ* ^2^(1) = 44.702, *p* < 0.001), with relatively more participants completing posttest in the WLC condition (141, 75.8%) than in the experimental condition (80, 41.9%). In the experimental condition, a significantly greater proportion of posttest completers (69, 86.3%) than dropouts (77, 69.4%) were in a stable relationship (*χ* ^2^(1) = 7.356, *p* = 0.007). In the control condition, a significantly greater proportion of posttest completers (45, 31.9%) than dropouts (7, 15.6%) were already practicing mindfulness (*χ* ^2^(1) = 4.533, *p* = 0.033), and posttest completers (*M* = 45.32, *SD* = 10.26) had a significantly higher average age than dropouts (*M* = 38.98, *SD* = 9.81) (*t*[184] = −3.646, *p* < 0.001). There were no significant associations or differences between posttest completers and dropouts in either condition on the other study variables at baseline.

**Fig. 1:**
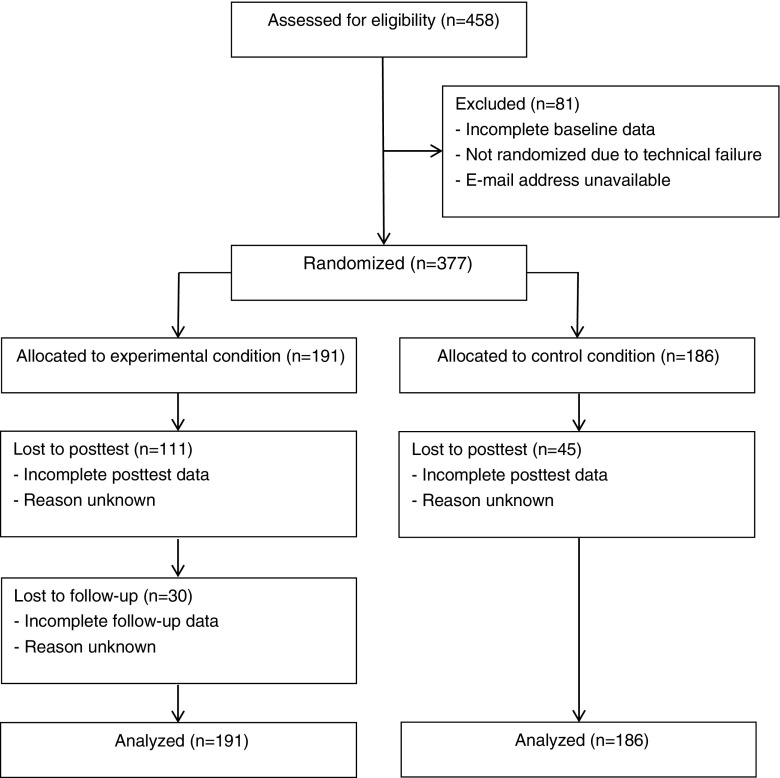
CONSORT diagram of participant flow through the study

Within the experimental condition, 50 (26.2%) of the 191 participants that were originally allocated to this condition completed the 3-month follow-up. Follow-up completers (*M* = 48.99, *SD* = 8.35) had a significantly higher average age than dropouts (*M* = 44.44, *SD* = 9.07) (*t*[189] = −3.106, *p* = 0.002). There were no significant associations or differences between follow-up completers and dropouts in this condition on the other study variables at baseline. Figure 1 displays the flow of participants through the study.

### Baseline Equivalence

There were no significant associations or differences between participants in the experimental and control conditions on the demographic variables at baseline or between the proportions of experimental and control participants that were currently practicing mindfulness or other forms of meditation (see Table 2). Similarly, there were no significant differences between experimental and control participants on the primary or secondary outcome measures at baseline (see Table 3, lowest *p* = 0.083).

**Table 2:** Demographic characteristics and meditation practices of participants in the experimental (*n* = 191) and WLC (*n* = 186) conditions

Study variable	Experimental	WLC	Test statistic
Age (Mean [*SD*])	45.63 (9.09)	43.78 (10.48)	*t*[375] = −1.831, *p* = 0.068
Sex (*n* [%])			
Male	8 (4.2)	7 (3.8)	*χ* ^2^(1) = 0.045, *p* = 0.833
Female	183 (95.8)	179 (96.2)	
Educational level (*n* [%])			
No or primary	13 (6.8)	10 (5.4)	*χ* ^2^(3) = 1.741, *p* = 0.628
Lower secondary	23 (12.0)	18 (9.7)	
Upper secondary	42 (22.0)	36 (19.4)	
Tertiary	113 (59.2)	122 (65.6)	
Relationship status (*n* [%])			
Single	45 (23.6)	48 (25.8)	*χ* ^2^(1) = 0.256, *p* = 0.613
In relationship	146 (76.4)	138 (74.2)	
Practicing mindfulness (*n* [%])			
Yes	50 (26.2)	52 (28.0)	*χ* ^2^(1) = 0.151, *p* = 0.697
No	141 (73.8)	134 (72.0)	
Practicing other meditation (*n* [%])			
Yes	45 (23.6)	34 (18.3)	*χ* ^2^(1) = 1.586, *p* = 0.208
No	146 (76.4)	152 (81.7)	

*WLC* waitlist control

**Table 3:** Baseline, posttest, and follow-up scores and Cohen’s *d* effect sizes for the experimental (*n* = 191) and WLC (*n* = 186) conditions

Study variable	Condition	Baseline mean (*SD*)	Posttest mean (*SD*)	Follow-up mean (*SD*)	Cohen’s *d*		
					Within-group baseline-posttest	Within-group baseline-follow-up	Between-group postttest
FFMQ-Total	Experimental	118.89 (19.33)	133.57 (17.95)	131.63 (22.11)	0.79***	0.61***	0.77***
	WLC	117.59 (17.65)	120.26 (19.14)		0.15*		
FFMQ-Observing	Experimental	24.91 (5.18)	28.76 (2.87)	28.79 (3.00)	0.92***	0.92***	0.66***
					0.13		
	WLC	24.71 (5.06)	25.36 (4.61)				
FFMQ-Describing	Experimental	27.27(6.47)	29.03 (4.30)	29.09 (3.48)	0.32***	0.35*	0.26*
	WLC	27.07 (6.09)	27.40 (5.41)		0.06		
FFMQ-Acting with awareness	Experimental	21.79 (5.53)	24.98 (3.75)	24.45 (3.52)	0.68***	0.57***	0.49***
	WLC	21.58 (5.08)	22.22 (4.73)		0.13		
FFMQ-Nonjudging	Experimental	24.77 (6.17)	27.24 (4.82)	27.29 (4.36)	0.45***	0.47***	0.34**
	WLC	24.58 (6.16)	25.11 (6.16)		0.09		
FFMQ-Nonreactivity	Experimental	20.16 (4.60)	23.08 (2.99)	23.14 (3.02)	0.75***	0.77***	0.43***
	WLC	19.66 (4.14)	20.72 (3.86)		0.27**		
WHOQOL-Physical health	Experimental	22.75 (3.85)	25.38 (4.85)	23.71 (3.77)	0.60***	0.25**	0.30
	WLC	22.18 (3.94)	23.72 (4.80)		0.35***		
WHOQOL-Psychological health	Experimental	18.26 (2.85)	20.90 (2.91)	19.14 (2.61)	0.92***	0.32***	0.38**
	WLC	17.74 (2.96)	19.32 (3.23)		0.51***		
WHOQOL-Social relationships	Experimental	10.27 (2.10)	11.21 (1.97)	10.45 (2.03)	0.46***	0.09	0.38**
	WLC	9.90 (2.31)	10.20 (2.31)		0.13*		
WHOQOL-Environment	Experimental	29.80 (3.62)	31.28 (3.08)	31.67 (3.20)	0.44***	0.55**	0.36*
	WLC	29.76 (3.54)	30.30 (3.58)		0.15*		
GHQ-12	Experimental	16.70 (6.74)	11.24 (6.01)	11.48 (5.76)	−0.86***	−0.83***	−0.68***
	WLC	16.82 (6.63)	15.63 (7.12)		−0.17*		
SISA	Experimental	40.14 (6.39)	42.84 (7.39)	41.70 (7.64)	0.39***	0.22***	0.10
	WLC	40.07 (6.20)	41.25 (6.61)		0.18**		

Posttest and follow-up means are based on the imputed values

*WLC* waitlist control, *FFMQ* Five Facet Mindfulness Questionnaire, *WHOQOL* World Health Organization Quality of Life, *SISA* Short Index of Self-Actualization

*p < 0.05; **p < 0.01; ***p < 0.001

### Outcome

Table 3 shows the imputed mean scores and corresponding Cohen’s *d* effect sizes for the pre- and posttest measurements. Online Resource 1 shows the observed (e.g., nonimputed) scores, which do not meaningfully differ from the imputed scores. On our primary outcome measure, we observed a significant interaction effect for the FFMQ total score, indicating superior performance of the VGZ Mindfulness Coach over the WLC condition, *b* = 11.89, *SE* = 1.94, *p* < 0.001. In addition, significant interaction effects in the same direction were observed for the FFMQ subscale scores (FFMQ-Observing, *b* = 3.27, *SE* = 0.54, *p* < 0.001; FFMQ-Describing, *b* = 1.26, *SE* = 0.59, *p* = 0.03; FFMQ-Acting with awareness, *b* = 2.95, *SE* = 0.59, *p* < 0.001; FFMQ-Nonjudging, *b* = 2.19, *SE* = 0.71, *p* < 0.01; FFMQ-Nonreactivity, *b* = 2.16, *SE* = 0.49, *p* < 0.001).

On our secondary outcome measures, we observed significant interaction effects for general psychiatric symptomatology (GHQ), *b* = 4.24, *SE* = 0.81, *p* < 0.001; psychological quality of life (WHOQOL-Psychological health), *b* = 1.03, *SE* = 0.35, *p* < 0.01; social quality of life (WHOQOL-Social relationships), *b* = 0.55, *SE* = 0.21, *p* < 0.01; and environmental quality of life (WHOQOL-Environment), *b* = 0.90, *SE* = 0.38, *p* < 0.05. For the following variables, we did not observe significant interaction effects: physical quality of life (WHOQOL-Physical), *b* = 0.71, *SE* = 0.49, *p* = 0.16, and self-actualization (SISA), *b* = 1.35, *SE* = 0.74, *p* = 0.07. We also ran the analyses based on the completer sample at posttest, and found that the same interaction effects reached significance with roughly the same between-group effect sizes (see Online Resource 2).

When correcting for multiple comparisons using the Bonferroni correction (dividing *p* = 0.05 by the number of outcome variables, i.e., *p*_corrected_ = 0.05/12 = 0.004), all significant interaction effects remained significant except for one subscale on the primary outcome measure (FFMQ-Describing, *p* = 0.03) and one subscale on one of the secondary outcome measures (WHOQOL-Social Relationships, *p* = 0.009). Of note, the interaction effects on the WHOQOL-Physical Health subscale (*p* = 0.16) and SISA (*p* = 0.07) were not significant in the original uncorrected analysis and are therefore not significant in the Bonferroni corrected analysis as well.

Table 2 also shows the imputed mean scores and corresponding Cohen’s *d* effect sizes for the follow-up measurement in the experimental condition. Compared to baseline, the magnitude of most effect sizes was smaller at follow-up than at posttest, except for environmental quality of life (WHOQOL-Environment) which showed a larger effect size at follow-up. Most within-group treatment effects were still significant at follow-up however. Specifically, on our primary outcome measure, we observed significant within-group effects for the FFMQ total score, *b* = 13.63, *SE* = 2.61, *p* < 0.001; FFMQ-Observing, *b* = 3.82, *SE* = 0.68, *p* < 0.001; FFMQ-Describing, *b* = 1.32, *SE* = 0.66, *p* < 0.05; FFMQ-Acting with awareness, *b* = 2.56, *SE* = 0.70, *p* < 0.001; FFMQ-Nonjudging, *b* = 2.68, *SE* = 0.76, *p* < 0.001; and FFMQ-Nonreactivity *b* = 3.03, *SE* = 0.60, *p* < 0.001.

With regard to our secondary outcome measures, within-group effects were significant at follow-up for general psychiatric symptomatology (GHQ), *b* = −6.26, *SE* = 1.00, *p* < 0.001; psychological quality of life (WHOQOL-Psychological health), *b* = 1.12, *SE* = 0.33, *p* < 0.001; environmental quality of life (WHOQOL-Environment), *b* = 1.88, *SE* = 0.43, *p* < 0.01; physical quality of life (WHOQOL-Physical health), *b* = 1.33, *SE* = 0.41, *p* < 0.01; and self-actualization (SISA), *b* = 2.85, *SE* = 0.79; *p* < 0.001. The only exception was that the follow-up treatment effect on social quality of life (WHOQOL-Social relationships) was not sustained at follow-up, *b* = 0.34, *SE* = 0.24, *p* = 0.15. Again, the same pattern was found for the completer sample at follow-up (see Online Resource 3).

### Satisfaction and Frequency of Use

Participants in the experimental condition who completed the posttest generally reported high satisfaction with all (partly overlapping) aspects of the VGZ Mindfulness Coach (see Table 4). On average, they reported to have used the app for a period of 3.64 weeks (*SD* = 1.34). Most participants (*n* = 56, 70.0%) reported to have used it “several times a week,” followed by nine participants (11.3%) who had used it “weekly,” “daily” (*n* = 6, 7.5%), or “occasionally” (5, 6.3%).

**Table 4:** Satisfaction data of participants in the experimental condition (*n* = 76)

	Mean (*SD*)^a^
I find the VGZ Mindfulness Coach comfortable to use	4.32 (0.79)
I find the mindfulness exercises useful	4.39 (0.80)
I am satisfied with the VGZ Mindfulness Coach	4.18 (0.84)
I find the background information on mindfulness useful	4.04 (0.94)
It is clear to me what I can do with the VGZ Mindfulness Coach	4.32 (0.91)
It is clear to me how I can follow the 5-week program	4.26 (0.99)
I like the voice-over of the mindfulness exercises	4.26 (1.11)
I would recommend the VGZ Mindfulness Coach to my friends or relatives	4.18 (0.95)
I find the mindfulness exercises easy to follow	4.29 (0.88)
I find the VGZ Mindfulness Coach clear and easy to understand	4.29 (0.91)
I find it clear what the exercises are about	4.47 (0.76)

^a^Range 1–5

Of the 50 participants in the experimental condition who completed the follow-up, 16 (32.0%) reported to still use the app at follow-up. Of these, seven participants (43.8%) reported to use it several times a week, followed by four participants (25.0%) who used it weekly, occasionally (*n* = 4, 25.0%), or “monthly” (*n* = 1, 6.3%).

None of these frequency-of-use indices was significantly related to changes of the FFMQ total or subscale scores at posttest or follow-up, while controlling for baseline and posttest scores, respectively (lowest *p* = 0.08; details available from the authors).

## Discussion

In this RCT, a self-help MBI app was associated with significant and substantial increases of mindfulness, as well as improvements of general psychiatric symptoms and psychological, social, and environmental quality of life. While most participants had discontinued or drastically reduced their use of the app at follow-up, the improvements were maintained for at least 3 months, although most effects were somewhat attenuated. An exception to this pattern was social quality of life, which had returned to baseline levels at follow-up. Satisfaction with the app was high.

Comparisons of our effect sizes to those in other primary outcome studies, let alone in meta-analyses, should be carefully interpreted given the differences in samples, outcome measures, interventions, and other study characteristics. With this in mind, our medium-to-large (Cohen 1988) between-group posttest effect size (*d* = 0.77) for mindfulness favorably compares to the effects of low-intensity self-help MBIs and MBIs in general. A recent review of such interventions (Cavanagh et al. 2014) included two studies that reported the effect of self-help MBIs on mindfulness measures (a comparison with the studies by Ly et al. (2014) and Howells et al. (2016) unfortunately was not possible since they did not report mindfulness scores, see “Introduction” section). Glück and Maercker (2011) compared a 2-week web-based mindfulness course to a WLC condition, and found a small-to-medium, but nonsignificant effect size favoring WLC (*g* = −0.31, 95% CI −0.88 to 0.26). In contrast, Morledge et al. (2013) compared an 8-week mindfulness-based stress management program to a no-intervention control group, and found a significant small-to-medium positive effect size favoring the intervention (*g* = 0.24, 95% CI 0.04 to 0.45). Interestingly, our effect size for mindfulness seems impressive even in light of the medium effect sizes for face-to-face MBIs in the meta-analyses of Eberth and Sedlmeier (2012) (*r* = 0.34) and Sedlmeier et al. (2012) (*r* = 0.28).

### Limitations

In designing this study, we placed much value on its external validity. Specifically, we tried to keep our screening and measurements as brief as possible to minimize the risk of measurement effects. Moreover, our eligibility criteria were limited to a few formal requirements that are not likely to have led to a selection bias. Inevitably, this focus on external validity contributed to a number of limitations that should be taken into account when interpreting our findings. First, our measurements relied on only self-report questionnaires, and while we controlled for prior experience with the practice of mindfulness and other forms of meditation, these potentially relevant background variables were measured in a limited way.

Second, the present app did not collect tracking data on how participants used and navigated through the app. We therefore had little control over our experimental manipulation and had to rely on self-reported data on frequency of use, which showed no relationships to changes in mindfulness. Of note, more objective adherence data did show such relationships in a recent meta-analysis of e-therapy studies. Specifically, this study found logins to be related to outcomes of physical health interventions and module completion to outcomes of psychological health interventions (Donkin et al. 2011).

Third, we had relatively high dropout rates and have no data on why participants discontinued their use of the app or their participation in the study. Such information might help future researchers to promote continued use of the app and to reduce study dropout. Of note is that our results were robust across the intention-to-treat versus completer analyses, suggesting that dropout did not meaningfully affect the present findings. Also, high dropout rates are not uncommon in e-health research and have even been described as “a natural and typical feature” rather than a limitation of such research (Eysenbach 2005, p. 1).

Fourth, the WHOQOL-BREF scales in our study showed poor (0.6 > *α* ≥ 0.5 for Psychological health) or questionable (0.7 > *α* ≥ 0.6 for Physical health, Social relationships, and Environment) internal consistency (George and Mallery 2003), which may have negatively affected our measurement of these quality-of-life aspects.

Fifth, although our study aimed to recruit a nonclinical population, the average GHQ-12 baseline scores were relatively high. To the extent that this limits the generalizability of our findings to healthy populations, it also underscores the potential of the VGZ Mindfulness Coach to reduce clinical symptoms or to prevent sub-clinical symptoms from reaching clinical thresholds. Evidence to date suggests, however, that MBIs and other meditation programs are not a panacea for all clinical outcome domains and may not exceed the efficacy of other active interventions (e.g., Goyal et al. 2014), and it is an empirical question whether the current results will generalize to different clinical samples.

Sixth, our sample was predominantly female. Although this appears to be the rule rather than the exception in similar studies (e.g., Demarzo et al. 2015; Howells et al. 2016; Ly et al. 2014) and in more or less adjoining research areas (e.g., Bower et al. 2013; Cuijpers et al. 2008), it limits the generalizability of our findings to male populations. The same is true for the comparatively high educational levels of our participants.

Seventh, our research team was not formally blinded to group allocation. Although we are confident that this has not influenced our analysis or findings, future studies should rule out this possibility by formally blinding the statistical analysis to group allocation.

Eighth, the study design does not allow us to disentangle a possible effect of the weekly e-mail reminders on frequency of app use or study adherence. For that, replication studies without these reminders are needed or studies with a comparison condition in which participants receive the app but not the reminders. Also, future studies should include placebo control conditions to rule out nonspecific effects as alternative explanations for the app’s efficacy.

In sum, despite these limitations, this study shows that it is possible to achieve durable positive effects on mindfulness, general psychiatric symptoms, and several aspects of quality of life at low costs with self-help MBIs such as the VGZ Mindfulness Coach app. We suggest that future studies remedy the above limitations (by, e.g., ensuring more equal sample distributions of sex and educational level, collecting objective adherence data, and including placebo control conditions) and explore the usefulness of MBI apps in specific clinical populations, for instance, as an adjunct to face-to-face treatments or as a means to prevent relapse after successful psychotherapies. Also, future research might now investigate the mechanisms that underlie the effects of the app, such as the possibility that improvements of psychiatric symptoms and quality of life are mediated by increased mindfulness. Of note, such research should take into account relationships of mindfulness with these and other psychosocial outcome variables may themselves be mediated by, e.g., prior meditation experience (Baer et al. 2008) or other variables.

## Electronic Supplementary Material

Supplemental Table S1 (DOCX 24 kb)

Supplemental Table S2 (DOCX 20 kb)

Supplemental Table S3 (DOCX 18 kb)
