# Supplementary material for: Efficacy of a Mindfulness-Based Mobile Application: a Randomized Waiting-List Controlled Trial
Source: Mindfulness (N Y). 2017 Jun 21;9(1):187–98. doi: 10.1007/s12671-017-0761-7 (PMC5770479; doi:10.1007/s12671-017-0761-7)
Supplement: Supplementary file 1 — (DOCX 24 kb) [file 12671_2017_761_MOESM1_ESM.docx]

*Supplemental Table S1.*

Observed Baseline, Posttest, and Follow-up Scores and Cohen’s *d* Effect Sizes for the Experimental and WLC Conditions

| Study variable | Condition | Baseline  Mean (*SD*) | Posttest  Mean (*SD*) | Follow-up  Mean (*SD*) | Cohen’s *d* | | |
| --- | --- | --- | --- | --- | --- | --- | --- |
|  |  |  |  |  | Within-group baseline-posttest | Within-group baseline-follow-up | Between-group postttest |
| FFMQ-Total | Experimental  WLC | 118.89 (19.33)  117.59 (17.65) | 133.13 (18.92)  120.74 (19.34) | 132.98 (20.91) | 0.74***  0.17* | 0.70*** | 0.75*** |
| FFMQ-Observing | Experimental  WLC | 24.91 (5.18)  24.71 (5.06) | 27.73 (3.81)  25.38 (5.15) | 28.85 (4.56) | 0.84***  0.13 | 0.81*** | 0.76*** |
| FFMQ-Describing | Experimental  WLC | 27.27(6.47)  27.07 (6.09) | 29.08 (5.99)  27.36 (6.06) | 29.15 (5.51) | 0.29***  0.05 | 0.31* | 0.34* |
| FFMQ-Acting with awareness | Experimental  WLC | 21.79 (5.53)  21.58 (5.08) | 25.04 (5.16)  22.19 (5.28) | 24.40 (5.48) | 0.61***  0.12 | 0.47*** | 0.60*** |
| FFMQ-Nonjudging | Experimental  WLC | 24.77 (6.17)  24.58 (6.16) | 27.24 (6.80)  25.11 (6.92) | 27.40 (7.07) | 0.38***  0.08 | 0.40*** | 0.35** |
| FFMQ-Nonreactivity | Experimental  WLC | 20.16 (4.60)  19.66 (4.14) | 23.05 (4.02)  20.70 (4.30) | 23.19 (4.55) | 0.67***  0.25** | 0.66*** | 0.49*** |
| WHOQOL-Physical health | Experimental  WLC | 22.75 (3.85) 22.18 (3.94) | 25.16 (5.29)  23.90 (4.79) | 23.98 (3.88) | 0.52***  0.39*** | 0.32** | 0.19 |
| WHOQOL-Psychological health | Experimental  WLC | 18.26 (2.85) 17.74 (2.96) | 21.05 (3.08)  19.45 (3.25) | 19.47 (2.59) | 0.94***  0.55*** | 0.44*** | 0.41** |
| WHOQOL-Social relationships | Experimental  WLC | 10.27 (2.10) 9.90 (2.31) | 11.25 (2.10)  10.21 (2.39) | 10.49 (2.00) | 0.47***  0.13* | 0.11 | 0.23** |
| WHOQOL-Environment | Experimental  WLC | 29.80 (3.62) 29.76 (3.54) | 31.33 (3.30)  30.46 (3.56) | 31.73 (3.27) | 0.44***  0.20* | 0.56** | 0.35* |
| GHQ-12 | Experimental  WLC | 16.70 (6.74) 16.82 (6.63) | 11.20 (6.47)  15.22 (7.15) | 10.38 (5.25) | -0.83***  -0.23* | -1.05*** | -0.63*** |
| SISA | Experimental  WLC | 40.14 (6.39) 40.07 (6.20) | 43.00 (7.59)  41.62 (6.78) | 42.84 | 0.41***  0.24** | 0.40*** | 0.13 |

*Note.* WLC = Waitlist Control. FFMQ = Five Facet Mindfulness Questionnaire. WHOQOL = World Health Organization Quality of Life. GHQ = General Health Questionnaire. SISA = Short Index of Self-Actualization. **p* < 0.05; ***p* < 0.01; ****p* < 0.001.
