# Supplementary material for: Efficacy of a Mindfulness-Based Mobile Application: a Randomized Waiting-List Controlled Trial
Source: Mindfulness (N Y). 2017 Jun 21;9(1):187–98. doi: 10.1007/s12671-017-0761-7 (PMC5770479; doi:10.1007/s12671-017-0761-7)
Supplement: Supplementary file 2 — (DOCX 20 kb) [file 12671_2017_761_MOESM2_ESM.docx]

*Supplemental Table S2.*

Baseline and Posttest Scores and Cohen’s *d* Effect Sizes for the Completers Sample of the Experimental (*n* = 80) and WLC (*n* = 141) Conditions

| Study variable | Condition | Baseline  Mean (*SD*) | Posttest  Mean (*SD*) | Cohen’s *d* | |  |
| --- | --- | --- | --- | --- | --- | --- |
|  |  |  |  | Within-group baseline-posttest | Between-group postttest | |
| FFMQ-Total | Experimental  WLC | 118.60 (18.36)  118.35 (17.41) | 133.13 (18.92)  120.74 (19.34) | 0.78***  0.13* | 0.82*** | |
| FFMQ-Observing | Experimental  WLC | 24.87 (4.82)  24.96 (4.86) | 28.73 (3.81)  25.38 (5.15) | 0.89***  0.08 | 0.79*** | |
| FFMQ-Describing | Experimental  WLC | 26.98 (5.90)  27.49 (6.60) | 29.08 (5.99)  27.36 (6.06) | 0.35***  0.02 | 0.27* | |
| FFMQ-Acting with awareness | Experimental  WLC | 21.85 (4.83)  21.64 (5.08) | 25.04 (5.16)  22.19 (5.28) | 0.64***  0.11 | 0.70*** | |
| FFMQ-Nonjudging | Experimental  WLC | 24.93 (6.28)  24.75 (7.01) | 27.24 (6.80)  25.11 (6.92) | 0.35***  0.05 | 0.42** | |
| FFMQ-Nonreactivity | Experimental  WLC | 19.72 (4.05)  19.76 (4.24) | 23.05 (4.02)  20.70 (4.30) | 0.82***  0.22*** | 0.61*** | |
| WHOQOL-Physical health | Experimental  WLC | 22.89 (4.29) 22.34 (3.86) | 25.16 (5.29)  23.90 (4.79) | 0.47***  0.36*** | 0.20 | |
| WHOQOL-Psychological health | Experimental  WLC | 18.46 (2.69) 17.88 (2.85) | 21.05 (3.08)  19.45 (3.25) | 0.90***  0.52*** | 0.39** | |
| WHOQOL-Social relationships | Experimental  WLC | 10.44 (2.20) 9.92 (2.41) | 11.25 (2.10)  10.21 (2.39) | 0.38***  0.12* | 0.11** | |
| WHOQOL-Environment | Experimental  WLC | 29.91 (3.77) 29.98 (3.53) | 31.33 (3.30)  30.46 (3.56) | 0.40***  0.14* | 0.57* | |
| GHQ-12 | Experimental  WLC | 16.75 (6.92) 16.34(6.67) | 11.20 (6.47)  15.22 (7.15) | -0.83***  -0.16* | -0.72*** | |
| SISA | Experimental  WLC | 40.37 (6.37) 40.36 (6.14) | 43.00 (7.59)  41.62 (6.78) | 0.38***  0.20** | 0.24 | |

*Note.* WLC = Waitlist Control. FFMQ = Five Facet Mindfulness Questionnaire. WHOQOL = World Health Organization Quality of Life. GHQ = General Health Questionnaire. SISA = Short Index of Self-Actualization. Completers are participants who filled out both the baseline and posttest measurements.

**p* < 0.05; ***p* < 0.01; ****p* < 0.001.
