# Supplementary material for: Efficacy of a Mindfulness-Based Mobile Application: a Randomized Waiting-List Controlled Trial
Source: Mindfulness (N Y). 2017 Jun 21;9(1):187–98. doi: 10.1007/s12671-017-0761-7 (PMC5770479; doi:10.1007/s12671-017-0761-7)
Supplement: Supplementary file 3 — (DOCX 18 kb) [file 12671_2017_761_MOESM3_ESM.docx]

*Supplemental Table S3.*

Baseline and Follow-up Scores and Cohen’s *d* Effect Sizes for the Completers Sample of the Experimental (*n* = 50) Condition

| Study variable | Baseline  Mean (*SD*) | Follow-up  Mean (*SD*) | Cohen’s *d* |
| --- | --- | --- | --- |
|  |  |  | Within-group baseline-follow-up |
| FFMQ-Total | 120.69 (16.80) | 135.20 (18.23) | 0.81*** |
| FFMQ-Observing | 25.42 (5.12) | 28.85 (4.56) | 0.71*** |
| FFMQ-Describinb | 28.08 (5.46) | 29.15 (5.51) | 0.20* |
| FFMQ-Acting with awareness | 21.89 (5.01) | 24.40 (5.48) | 0.48*** |
| FFMQ-Nonjudging | 24.66 (6.52) | 27.40 (7.07) | 0.40*** |
| FFMQ-Nonreactivity | 20.26 (4.41) | 23.19 (4.55) | 0.65*** |
| WHOQOL-Physical health | 22.68 (4.27) | 24.16 (3.69) | 0.37*** |
| WHOQOL-Psychological health | 18.44 (2.65) | 19.48 (2.61) | 0.40** |
| WHOQOL-Social relationships | 10.12 (2.14) | 10.52 (2.01) | 0.19 |
| WHOQOL-Environment | 29.94(3.50) | 31.76 (3.29) | 0.54*** |
| GHQ-12 | 16.82 (7.57) | 11.48 (5.76) | -0.99*** |
| SISA | 39.82 (5.51) | 42.88 (7.29) | 0.47*** |

*Note.* WLC = Waitlist Control. FFMQ = Five Facet Mindfulness Questionnaire. WHOQOL = World Health Organization Quality of Life. GHQ = General Health Questionnaire. SISA = Short Index of Self-Actualization.

**p* < 0.05; ***p* < 0.01; ****p* < 0.001. Completers are participants who filled out both the

baseline and follow-up measurements.
